# *N*-Glycosylation Facilitates 4-1BB Membrane Localization by Avoiding Its Multimerization

**DOI:** 10.3390/cells11010162

**Published:** 2022-01-04

**Authors:** Ruoxuan Sun, Alyssa Min Jung Kim, Allison A. Murray, Seung-Oe Lim

**Affiliations:** 1 Department of Medicinal Chemistry and Molecular Pharmacology, Purdue University, West Lafayette, IN 47907, USA; sun714@purdue.edu (R.S.); kim1705@purdue.edu (A.M.J.K.); murra185@purdue.edu (A.A.M.); 2 Purdue Institute of Drug Discovery, Purdue University, West Lafayette, IN 47907, USA; 3 Purdue Center for Cancer Research, Purdue University, West Lafayette, IN 47907, USA

**Keywords:** 4-1BB, *N*-glycosylation, degradation, oligomerization

## Abstract

Leveraging the T cell immunity against tumors represents a revolutionary type of cancer therapy. 4-1BB is a well-characterized costimulatory immune receptor existing on activated T cells and mediating their proliferation and cytotoxicity under infectious diseases and cancers. Despite the accumulating interest in implementing 4-1BB as a therapeutic target for immune-related disorders, less is known about the pattern of its intracellular behaviors and regulations. It has been previously demonstrated that 4-1BB is heavily modified by *N*-glycosylation; however, the biological importance of this modification lacks detailed elucidation. Through biochemical, biophysical, and cell-biological approaches, we systematically evaluated the impact of *N*-glycosylation on the ligand interaction, stability, and localization of 4-1BB. We hereby highlighted that *N*-glycan functions by preventing the oligomerization of 4-1BB, thus permitting its membrane transportation and fast turn-over. Without *N*-glycosylation, 4-1BB could be aberrantly accumulated intracellularly and fail to be sufficiently inserted in the membrane. The *N*-glycosylation-guided intracellular processing of 4-1BB serves as the potential mechanism explicitly modulating the “on” and “off” of 4-1BB through the control of protein abundance. Our study will further solidify the understanding of the biological properties of 4-1BB and facilitate the clinical practice against this promising therapeutic target.

## 1. Introduction

The pharmacological targeting of immune checkpoint molecules has redefined the paradigm of cancer therapy [1,2]. T cells, commonly considered as the key players that dictate anti-tumor immunity, are regulated by a variety of co-stimulatory and co-inhibitory receptors to preserve their fine-tuning under immune reactions [3,4,5]. 4-1BB (CD137/TNFRSF9), a type I transmembrane glycoprotein, is known as a T cell-associated molecule that is required for sufficient T cell immunity against cancer and infectious diseases [6,7,8]. The expression of 4-1BB can be induced by either T cell activation or hypoxia [9,10]. Systematic loss of 4-1BB results in compromised immune function due to the rendered T cell cytotoxicity and cytokine production [11]. 4-1BB ligand (4-1BBL/CD137L/TNFSF9) is the only cognate ligand that has been identified so far. Like other members of the tumor necrosis factor receptor (TNFR) superfamily, 4-1BB is activated when crosslinked by trimeric 4-1BBL on antigen-presenting cells [12,13]. The signal transduction of crosslinked 4-1BB relies on the recruitment of TNFR-associated-factor (TRAF) family adaptor proteins to its cytoplasmic tail, followed by signaling through NF-kB, which acts as the master transcription factor driving the expression of pro-survival and T cell cytotoxicity genes [13,14]. Targeting of 4-1BB using agonistic antibodies elicits robust immune activation effects, which can be applied to cancer therapy [15,16]. The clinical development of two 4-1BB agonistic antibodies, namely urelumab and utomilumab, is ongoing for anti-tumor purposes [17]. It has also been reported that the secreted form of 4-1BB, which is generated through either alternative mRNA splicing or cleavage by membrane-bound metalloproteases [18,19], diminishes the 4-1BB/4-1BBL axis by competing against the T cell functional surface 4-1BB.

The precise regulation of many proteins depends on the post-translational modifications (PTM) they underwent. Amongst over 400 kinds of identified PTMs [20], *N*-linked glycosylation is one of the most complicated and heterogeneous forms [21,22,23]. Protein *N*-glycosylation, by definition, is the process whereby carbohydrate chains are covalently attached to substrates, and it is involved in a wide range of cellular processes including protein folding, transportation, stability, protein–protein interactions, etc. [24,25]. Dysregulated protein *N*-glycosylation is associated with many diseases, including, but not limited to, cancer and immune disorders [26,27]. Protein *N*-glycosylation is a highly conserved sequential reaction in eukaryotic systems. In the endoplasmic reticulum (ER), an oligosaccharide chain is attached to the consensus N-X-S/T (-Asn-X-Ser/Thr-) motif of the substrate [21,22,23]. Subsequently, in the Golgi apparatus, various enzymes add, trim, or branch the existing glycan chains, resulting in various arrangements of sugar moieties with increased complexities. At this point, the glycosylated protein is fully synthesized and will be delivered to its corresponding subcellular compartments [21,22,23].

Glycosylation plays essential and comprehensive roles in the immune system [28,29], and one of which is to functionally regulate the immune receptors [30]. Our group has shown that human 4-1BB can be modulated by *N*-glycosylation [31]; however, the underlying molecular function of this modification has yet to be interrogated. In the present research, we aimed to explore whether *N*-glycosylation affects the ligand binding, stability, and subcellular compartmentalization of 4-1BB. While the ligand binding of 4-1BB appears to be glycosylation-independent, this protein relies on its *N*-link glycans to achieve its membrane localization. Without *N*-glycosylation, 4-1BB is retained within the cells and preferentially forms stable multimers, which cannot be delivered to cell surfaces efficiently. In summary, the more profound elucidation of glycosylation, as well as other unique modifications of 4-1BB, may help to advance immunotherapeutic drug development in the future.

## 2. Materials and Methods

### 2.1. Cell Culture

HEK293T cells were purchased from Takara Bio (San Jose, CA, USA) and maintained in DMEM medium (Gibco, Waltham, MA, USA) with 10% fetal bovine serum (FBS, Corning, Oneonta, NY, USA) and penicillin-streptomycin (pen/strep, Gibco). Jurkat cells were obtained from American Type Culture Collection (Manassas, VA, USA) and cultured in RMPI1640 medium (Cytiva, Marlborough, MA, USA), supplemented with 10% FBS, 10 mM HEPES, and pen/strep. All cells were kept in humified incubators with 5% CO_2_ at 37 °C.

### 2.2. Plasmid and Lentivirus

The cDNA of human 4-1BB (Sino Biological, Wayne, PA, USA) was inserted into pcDNA3, pGIPZ, and pHR-SFFV vectors by restriction cloning (NheI/NotI) or HiFi assembly (New England BioLabs, Ipswich, MA, USA) for transient or stable expression in HEK293T or Jurkat cells. All single mutations were achieved using a Q5 site-directed mutagenesis kit (New England BioLabs). The sequences of all constructs were confirmed by Sanger sequencing (Genewiz, South Plainfield, NJ, USA) before use. X-TremeGENE HP (Roche, Indianapolis, IN, USA) was used for transient DNA transfection (DNA:Transfection reagent = 1:2.5). To produce lentivirus for stable gene expression, pMD2.G, psPAX2, and lentiviral constructs were co-transfected at a ratio of 1:1.5:2 to HEK293T cells. The supernatant containing lentivirus particles was collected 48 h after transfection. For transduction of Jurkat cells, the lentivirus (made with pHR-SFFV vector) was incubated with cells for 16 h and protein expression was confirmed three days after transduction by either flow cytometry or immunoblotting. To transduce HEK293T cells, the lentivirus (made with pGIPZ vector) was added to cell culture with 8 μg/mL polybrene (Santa Cruz, Dallas, TX, USA), incubated overnight, and followed by addition of 2 μg/mL puromycin (Invivogen, San Diego, CA, USA) for selection.

### 2.3. Binding Affinity Detection by Enzyme-Linked Immunoassay (ELISA)

ELISA was performed to compare the receptor/ligand and receptor/antibody binding. To get *N*-glycan-abolished 4-1BB protein, His-tagged 4-1BB protein (Sino Biological, expressed in HEK293 cells) was treated with PNGase F (New England BioLabs) under non-reducing conditions, following the manufacturers’ instruction. The digestion was validated by Coomassie blue staining. Native and deglycosylated 4-1BB were loaded on a Ni-NTA-coated 96-well plate and incubated at room temperature for 30 min. After three washes with PBST (phosphate-buffered saline (PBS)/0.1% Tween-20), human Fc-tagged 4-1BBL (Sino Biological, expressed in HEK293 cells, diluted in PBST) or mouse anti-human 4-1BB antibody (4B4-1, 1:200, BioLegend # 309802, San Diego, CA, USA) was added and incubated for another 30 min, followed by three washes. Plate-bound 4-1BBL or 4B4-1 was incubated with horseradish peroxidase-conjugated anti-human/mouse Fc secondary antibody (Invitrogen, Waltham, MA, USA) and developed with TMB substrate (Cell signaling technology, Danvers, MA, USA). The quantified result was acquired on a Synergy LX microplate reader (BioTek, Winooski, VT, USA) by measuring the OD_450_ value.

### 2.4. Binding Affinity (K_D_) Determination

The binding affinity (K_D_) of 4-1BB/4-1BBL was determined by Octet Biolayer interferometry (BLI) using the Octet RED384 system (Sartorius, Bohemia, NY, USA). Briefly, the native and deglycosylated His-tagged 4-1BB proteins (prepared as described in Section 2.3) were loaded on the Octet NTA biosensor at a concentration of 200 nM. The association step was performed by submerging the sensors in a single concentration of 4-1BBL (50 mM) in the kinetic buffer. Dissociation was performed and monitored in fresh kinetic buffer. Data were analyzed with Octet Analysis HT software (Sartorius). The K_D_ value was calculated based on a 1:1 binding ratio.

### 2.5. Immunoblotting and In Vivo Ubiquitination Assay

For immunoblotting analysis, cells were lysed in ice-cold RIPA buffer (50 mM Tris-HCl, pH 7.5, 150 mM NaCl, 1% NP-40, 0.1% SDS, 1 mM EDTA, 0.5% sodium deoxycholate) and heated with 2-mercaptoethanol (2-ME). Samples were normalized by BCA assay kit (Pierce, Waltham, MA, USA), separated on sodium dodecyl sulfate–polyacrylamide gel electrophoresis (SDS-PAGE), and transferred to PVDF membrane (Millipore, St. Louis, MO, USA), followed by blocking in 5% skim milk (Research Products International, Mt Prospect, IL, USA). To detect protein oligomerization, samples were supplemented both with and without 50 mM dithiothreitol (DTT) before SDS-PAGE running. Protein ubiquitination was analyzed by in vivo ubiquitination assay. Briefly, cells were pre-treated with 20 μM MG-132 (Selleck, Houston, TX, USA) for 6 h, lysed in NP-40 buffer (50 mM Tris-HCl, pH 7.5, 150 mM NaCl, 1% NP-40, 1 mM EDTA), incubated with 1% SDS at 95 °C for 10 min, and diluted 10-fold with NP-40 buffer to allow immunoprecipitation with anti-Flag agarose (Sigma Aldrich #A2220) overnight. After four intense washes with RIPA buffer, the immunoprecipitated proteins were eluted in Laemmli sample buffer (Bio-Rad, Hercules, CA, USA) with 2-ME. The following steps for sample analysis were performed identically to immunoblotting above. The following primary antibodies and antibody-conjugated beads were used for overnight incubation with blots: Flag-tag (Sigma Aldrich #F1804, 1:1000, mouse), DYKDDDDK-tag (Cell signaling technology #2368, 1:2000, rabbit, binds to the same epitope as Flag-tag antibody), β-actin (Santa Cruz #sc-47778, 1:2000, mouse), GAPDH (Cell signaling technology #5174, 1:3000, rabbit), HA-tag (Cell signaling technology #3724. 1:2500, rabbit), 4-1BB (Cell signaling technology #34594, 1:3000, rabbit), and GFP (Santa Cruz #sc-9996, 1:2000, mouse). Subsequently, the membranes were incubated with IRDye-conjugated secondary antibodies (1:20,000, LI-COR Biosciences, Lincoln, NE, USA) and visualized on Odyssey CLx imager (LI-COR Biosciences). Image processing and analysis were done with Image Studio software (version 5.0).

### 2.6. Flow Cytometry Analysis

Jurkat cells were washed twice with ice-cold staining buffer (PBS/0.5% BSA/2 mM EDTA) and stained with APC-conjugated anti-4-1BB antibody (BD #561702) for 30 min at 4 °C. After three washes with staining buffer, cell samples were loaded on an Attune NXT flow cytometer (Invitrogen) for analysis. Data analysis was performed on Attune NxT software.

### 2.7. Cycloheximide (CHX)-Chase Assay

HEK293T cells were transiently transfected with plasmids and then trypsinized and transferred into multi-well plates for overnight culture. The de novo protein synthesis inhibitor CHX (ACROS Organics, Pittsburgh, PA, USA) was added at a concentration of 100 uM to cells and incubated for 0–8 h. The total cellular protein samples were harvested at each time point for later immunoblotting assay (described above). The relative intensities of bands were normalized against β-actin, and 0 h samples were considered 100%.

### 2.8. N-Glycomic Analysis of Human 4-1BB

*N*-glycomic analysis of the human 4-1BB protein was performed by methods described previously [32]. Briefly, *N*-glycans of HEK293-expressed 4-1BB protein (Sino Biological) were released by treating the reduced and alkylated protein with PNGase F. The released *N*-glycan fractions were then permethylated. The permethylated *N*-glycans were evaluated by matrix-assisted laser desorption/ionization—mass spectrometry (MALDI-MS) using the AB SCIEX TOF/TOF 5800 mass spectrometer (Applied Biosystem/MDS Analytical Technologies, Waltham, MA, USA). The structural assignments of the *N*-glycans were based on molecular weight and followed the principles of the *N*-glycan bio-synthesis pathway. The carbohydrate analysis was performed at the Complex Carbohydrate Research Center, the University of Georgia (supported by NIH R24GM137782 grant).

### 2.9. Statistics

All quantitative results were displayed as the mean ± SD, with at least three biological replicates. The inter-group statistical significance was calculated by two-tail Student’s *t*-test. *p* < 0.05 was considered statistically significant.

## 3. Results

### 3.1. 4-1BB Is N-Glycosylated on Two Conserved N-X-S/T Motifs

**Figure 1:**
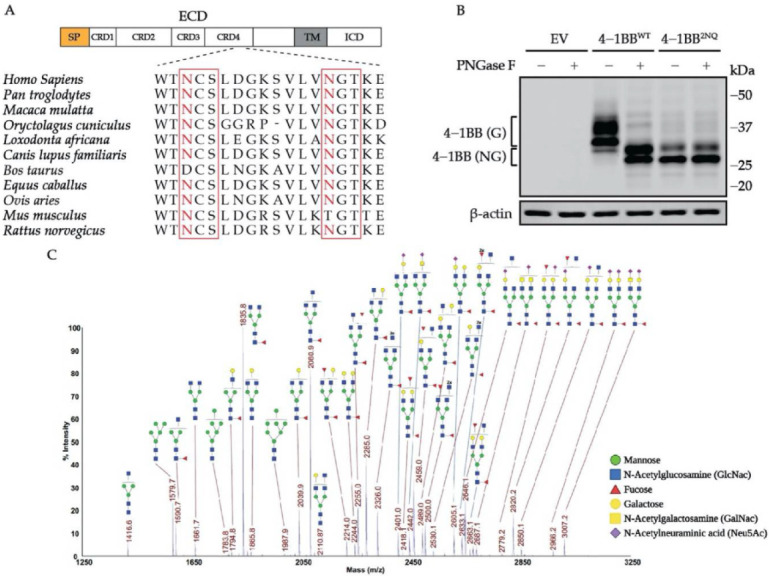
4-1BB is a heavily *N*-glycosylated protein with two modified sites. (**A**) Alignment of mammalian 4-1BB sequence around the *N*-glycosylation sites. The N-X-S/T motif is outlined by the red square. (**B**) Immunoblotting analysis of 4-1BB undergoing deglycosylation treatment by PNGase F. (**C**) MALDI-MS profiling of permethylated N-glycans released from PNGase F-treated recombinant 4-1BB protein. The masses of indicated glycan species represents the [M + Na^+^] values. SP, signal peptide; ECD, extracellular domain; TM, transmembrane domain; ICD, intracellular domain; CDR, cysteine-rich domain; EV, empty vector; 4-1BB (G), *N*-glycosylated 4-1BB; 4-1BB (NG), non-*N*-glycosylated 4-1BB.

Canonical *N*-glycosylation occurs on N-X-S/T motifs of substrates [21,22,23]. We first examined the amino acid sequence of mammalian 4-1BB proteins and identified two conserved, extracellular domain-localized N-X-S/T motifs that could potentially be *N*-glycosylated, namely N138 and N149 (based on human sequence, shown in Figure 1A). To verify the *N*-glycosylation status of native 4-1BB, we established HEK293T cell lines stably overexpressing wild-type (WT) or N138Q/N149Q (2NQ) mutation and treated the cell lysates with PNGase F to study the enzymatic deglycosylation effect. As illustrated by the immunoblotting result in Figure 1B, WT protein showed an increased mobility shift after PNGase F digestion. On the contrary, the 2NQ mutation protein no longer responds to the treatment, indicating that human 4-1BB only possesses these two *N*-glycosylation sites. We also performed glycomics analysis to profile the various *N*-glycan species on 4-1BB. The HEK293T cell-expressed human 4-1BB protein that has intact *N*-glycosylation was used for glycan profiling. Over 30 types of *N*-linked glycans derived from the two modified sites can be identified by MALDI-TOF mass spectrometry (Figure 1C and Table S1), thus demonstrating the extensive heterogeneity of *N*-glycosylation on 4-1BB. These results verified that 4-1BB protein is *N*-glycosylated on two N-X-S/T motifs exclusively and appears as a diverse pool of glycoforms.

### 3.2. N-Glycosylation of Human 4-1BB Has Minimal Contribution to the Binding with 4-1BBL

**Figure 2:**
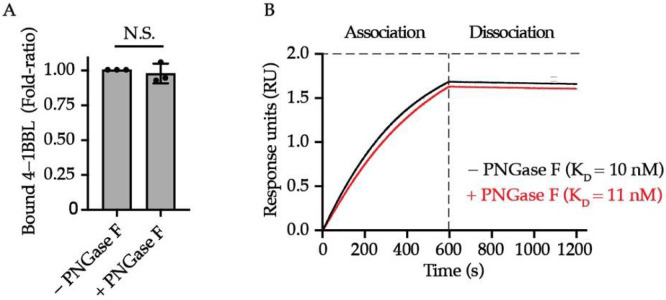
The impact of *N*-glycosylation on 4-1BB/4-1BBL interaction. (**A**) The relative level of bound 4-1BBL to PNGase F-treated and untreated 4-1BB protein (*n* = 3). (**B**) Octet analysis calculating the binding affinity of glycosylated (black) and deglycosylated 4-1BB (red) to 4-1BBL along with the K_D_ values. N.S., not significant (Two-tailed student’s *t*-test).

One well-characterized function of protein *N*-glycan moieties is to mediate the ligand/antibody binding to immune receptors such as natural killer cell receptor 2B4 (2B4), inducible co-stimulator (ICOS), programmed cell death 1 ligand (PD-L1), etc. [33,34,35,36]. As unveiled by the structural biology works, 4-1BB interacts with 4-1BBL through cysteine-rich domains (CRD) 2 and 3 [37,38], which are distant from the two *N*-glycosylation sites located on CRD4, leading us to the assumption that 4-1BB/4-1BBL engagement is likely to be *N*-glycosylation independent. To test this hypothesis, we performed both ELISA and Octet-based BLI assay to quantitively compare the binding affinity and kinetics of 4-1BBL to 4-1BB with intact or ablated *N*-glycosylation. The PNGase F-catalyzed deglycosylation of 4-1BB was confirmed by Coomassie blue staining (Figure S1). As unveiled by ELISA results, the binding capacity of 4-1BB to its ligand is *N*-glycosylation independent (Figure 2A). Octet assay results in Figure 2B also confirmed that the KD values between PNGase F-untreated and -treated 4-1BB (10 nM and 11 nM, respectively) are similar. Collectively, we concluded that *N*-linked glycan is not a prerequisite for 4-1BB to interact with 4-1BBL. 

### 3.3. N-Glycosylation Contributes to the Transportation of 4-1BB to Membrane

**Figure 3:**
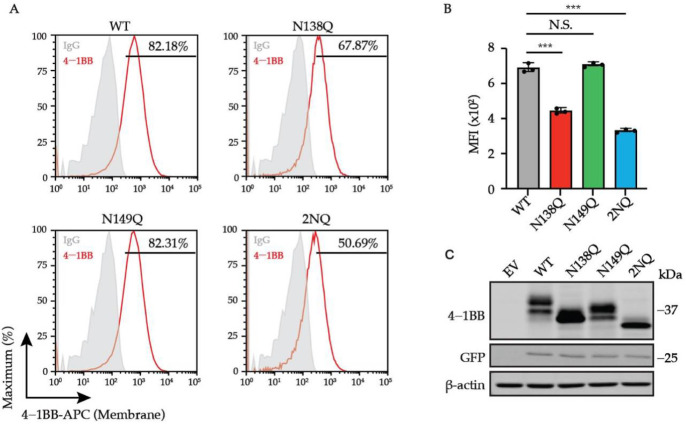
The regulation of cell surface level of 4-1BB by *N*-glycosylation. (**A**) Jurkat cells were transduced with lentivirus encoding 4-1BB WT and glycosylation-deficient mutant including N138Q, N149Q and 2NQ, respectively. The membrane levels of 4-1BB in each group were compared by flow cytometry. The percentages of positive events were included in the plots. (**B**) The quantitative results of membrane 4-1BB level in each group in (**A**) (*n* = 3). (**C**) The immunoblotting analysis of total 4-1BB from cells in (**A**). N.S., not significant; ***, *p* < 0.001 (Two-tailed student’s *t*-test); MFI, mean fluorescence intensity.

*N*-glycosylation is required for the membrane trafficking of many membrane proteins. To evaluate the impact of *N*-glycans on 4-1BB membrane expression, we established a panel of 4-1BB mutants with single or double mutation of its glycosylated asparagine sites (N138 and N149) and compared their total and membrane levels on Jurkat cells by executing flow cytometry and immunoblotting assays. The lentivirus vector with spleen focus-forming virus (SFFV) promoter was chosen because it promotes robust gene expression in hematopoietic cells [39]. A T2A-EGFP cassette was fused downstream of the 4-1BB coding region as a reporter monitoring the dose of lentivirus transduction (Figure S2). As demonstrated in Figure 3A–C, the single mutation of N138Q, but not N149Q, led to a notable decrease of membrane 4-1BB level without affecting the overall protein expression. Double mutation of the two sites (designated as 2NQ) further reduced 4-1BB membrane expression compared to N138Q alone. Additionally, according to immunoblotting analysis, the N138Q mutation caused more molecular weight decrease than N149Q, indicating that N138 was used to form a larger glycan architecture, which potentially harbors more impactful functions. We also confirmed that the antibody we utilized for flow cytometry staining (clone 4B4-1) does not distinguish glycosylated non-glycosylated 4-1BB in respect of binding (Figure S3), thus ensuring that the decreased membrane levels of glycosylation-deficient mutants were caused by the alteration of the protein itself. Hence, we proposed that *N*-glycosylation plays a role in transporting 4-1BB onto the cell membrane. 

### 3.4. 4-1BB Is Destabilized by N-Glycosylation

**Figure 4:**
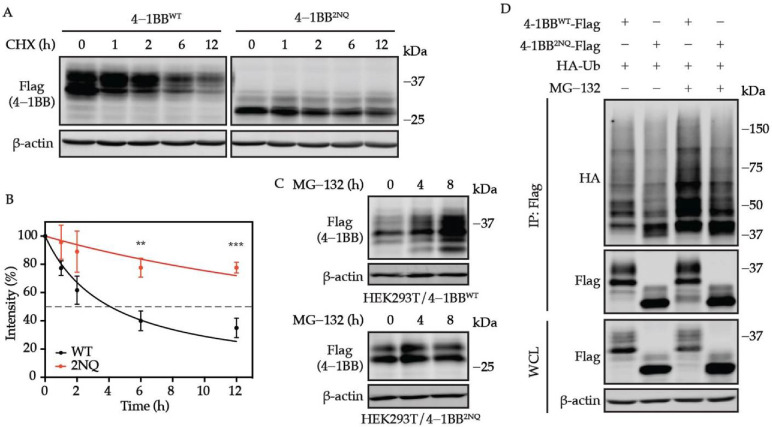
*N*-glycosylation plays an essential role in determining 4-1BB stability. (**A**) HEK293T cells were transfected with Flag-tagged 4-1BB WT and 2NQ followed by CHX-chase assay to compare their stability. (**B**) The quantification of the remaining 4-1BB at each time point (*n* = 3). The percentage values of band intensity were normalized to time 0 h. 50% degradation is indicated by the dashed line. (**C**) Immunoblotting analysis of the MG-132 effect on 4-1BB WT and 2NQ. HEK293T/4-1BB WT and HEK293T/4-1BB 2NQ cells were treated by 10 μM MG-132 for 0, 4, and 8 h followed by immunoblotting assay. (**D**) Comparison of polyubiquitination level between WT and 2NQ 4-1BB. Flag-tagged 4-1BB and HA-tagged Ub were co-transfected to HEK293T cells followed by in vivo ubiquitination assay 48 h after transfection. IP, immunoprecipitates. **, *p* < 0.01; ***, *p* < 0.001 (Two-tailed student’s *t*-test).

One of the best-characterized functions of *N*-glycosylation is to regulate protein stability [24,25]. Therefore, we compared the degradation half-life of 4-1BB WT and 2NQ to study the role *N*-glycan plays in the turn-over of 4-1BB. Interestingly, 4-1BB displayed dramatically increased stability in CHX-chase assay when lacking *N*-glycosylation sites, which is the opposite of many reported glycosylated immune receptors [30] (Figure 4A,B). Unlike the WT, which was markedly upregulated upon proteasome inhibition, the 2NQ mutation exerted only mild accumulation upon the treatment of proteosome inhibitor MG-132 (Figure 4C), suggesting that ubiquitination targets 4-1BB for degradation. As demonstrated in Figure 4D, the 2NQ mutation of 4-1BB showed a decreased level of polyubiquitination compared to WT. Collectively, we concluded that *N*-glycosylation contributes to the rapid degradation of 4-1BB through the ubiquitin-proteasome pathway. According to our unpublished data, 4-1BB is ubiquitinated on its intracellular domain, which guides its proteasomal degradation. Some E3 ubiquitin ligases are known to ubiquitinate the glycosylated substrates by recognizing the *N*-glycan moieties [40]; however, since the extracellular *N*-linked oligosaccharide chains and the intracellular ubiquitination sites are separated by plasma membranes, the decreased ubiquitination observed on 2NQ is unlikely to be caused by insufficient E3 ligase binding out of glycan loss. Therefore, we investigated the potential mechanisms to explain the vastly increased stability of non-*N*-glycosylated 4-1BB.

### 3.5. N-Glycosylation-Deficient 4-1BB Is More Intensively Multimerized

**Figure 5:**
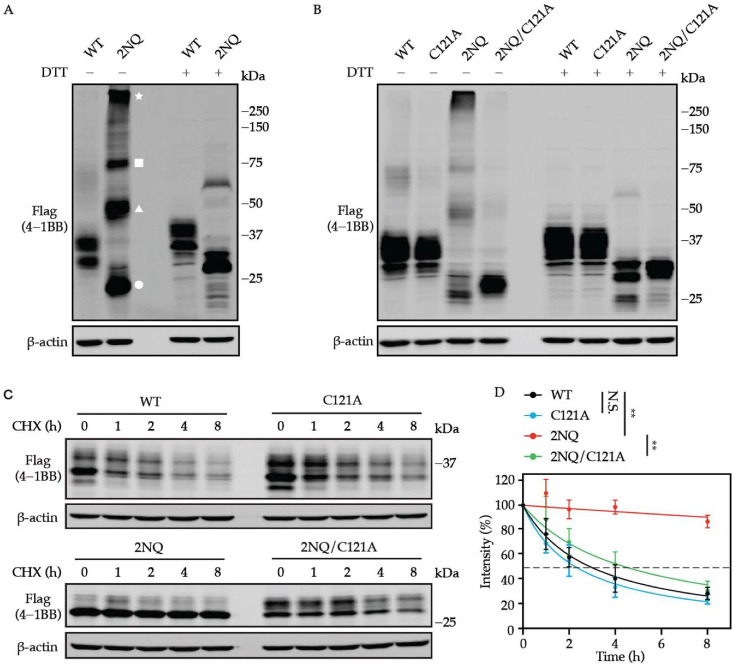
Oligomerization is associated with augmented stability of unglycosylated 4-1BB. (**A**) Non-reducing SDS-PAGE uncovered the formation of dimerized, trimerized, and oligomerized 4-1BB 2NQ. The bands that exist in the presence of DTT were considered monomers. The white circle, triangle, square, and star represent the monomerized, dimerized, trimerized, and oligomerized form of 4-1BB 2NQ. (**B**) 4-1BB 2NQ failed to form multimers when lacking the key cysteine residue C121. (**C**) HEK293T cells were transfected with Flag-tagged 4-1BB WT, C121A, 2NQ, and 2NQ/C121A followed by CHX-chase assay to compare their stability. (**D**) The quantification of the remaining 4-1BB at each time point (*n* = 3). The percentage values of band intensity were normalized to time 0 h. 50% degradation is indicated by the dashed line. Two-tailed student’s t-test was performed to compare the values on 8 h point. N.S., not significant; **, *p* < 0.01.

Protein oligomerization could be regulated by *N*-glycosylation [41,42]. Thus, we sought to examine whether the *N*-glycans present affect the multimer formation of 4-1BB. By performing non-reducing SDS-PAGE, we noticed substantially increased oligomerization was formed by non-glycosylated 4-1BB but not WT (Figure 5A). The ectodomain of 4-1BB is composed of four CRDs, which are the hallmark structures of the TNFRSF receptor family [43]. As illustrated by previous structural investigations [37,38], 20 out of 21 cysteine residues in the extracellular domain are used to form intramolecular disulfide bonds, which maintain the structural integrity of human 4-1BB. C121, the only unpaired cysteine, which is found in CRD4, can be used to create an intermolecular disulfide bond to stabilize 4-1BB dimers. As displayed in Figure 5B, the oligomerization of 4-1BB 2NQ was largely diminished when a cysteine-to-alanine mutation was introduced to C121. Likewise, the slow turn-over of 4-1BB 2NQ was also rescued by the C121A mutation (Figure 5C,D). 

**Figure 6:**
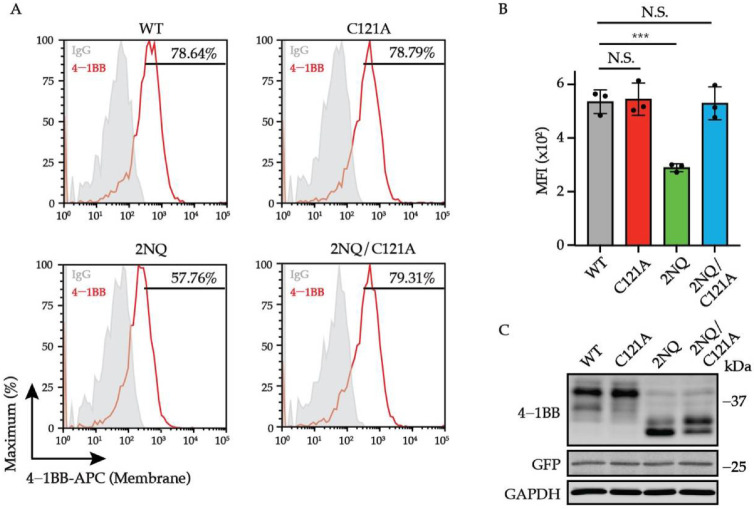
C121-mediated multimerization governs the stability and localization of unglycosylated 4-1BB. (**A**) Jurkat cells were transduced with lentivirus encoding 4-1BB WT, C121A, 2NQ, and 2NQ/C121A, respectively. The membrane levels of 4-1BB in each group were compared by flow cytometry. The percentages of positive events were included in the plots. (**B**) The quantitative results of membrane 4-1BB level in each group in (**A**) (*n* = 3). (**C**) The immunoblotting analysis of total 4-1BB from each group of cells in (**A**). N.S., not significant; ***, *p* < 0.001 (Two-tailed student’s *t*-test); MFI, mean fluorescence intensity.

To further study whether the turn-over of 4-1BB is associated with membrane transportation, we compared the surface level of 4-1BB 2NQ with that of 4-1BB 2NQ/C121A. As shown in Figure 6A,B, in the absence of the C121-mediated disulfide bond, *N*-glycosylation-deficient 4-1BB exhibited comparable expression to WT, which is much higher than 2NQ. The overall expression of 4-1BB 2NQ and 2NQ/C121A are similar, despite the altered ratio of the two bands. By integrating the observations above, we proposed the model whereby *N*-glycosylation of 4-1BB hinders the C121-mediated multimerization, therefore allowing 4-1BB to be correctly delivered via the secretory pathway to the cell membrane, where it undergoes ubiquitination-triggered degradation.

## 4. Discussion

Glycosylation refers to the conjugation of carbohydrate chains to predominantly protein substrates, but also lipids, and even nucleic acids [27,44]. *N*-linked glycosylation is a frequently occurring, post-translational modification of proteins in the context of immune receptors. Its enormous complexity makes the site-specific dissection and manipulation of protein *N*-glycosylation extremely challenging [45]. The predicted molecular weight of human 4-1BB (with C-terminus Flag tag) without signal peptide is about 26.3 kDa, which is far lower than the molecular weights of two major bands observed on SDS-PAGE (approximately 32 and 40 kDa), thus suggesting significant structural, and potentially functional, contributions of *N*-glycan to 4-1BB. 

Our study is the first report focusing on the biological value of *N*-glycosylation to the immune activation receptor 4-1BB. Specifically, human 4-1BB undergoes *N*-glycosylation on N138 and N149 for its maturation and membrane localization, and the glycan moieties indeed caused the molecular weight increase compared to the theoretical value. Mechanistically, once the *N*-linked oligosaccharide chains are no longer conjugated to 4-1BB, the under-glycosylated protein will alternatively use its free cysteine residue, C121, to form disulfide bond-mediated dimers and oligomers, which become considerably more stable than monomers and consequently cannot be appropriately trafficked towards the cell membrane. Nonetheless, several questions remain to be addressed. Given that *N*-glycosylation serves as a quality control step in protein synthesis and folding, and abnormal glycan structures can mark the protein for ER-associated degradation (ERAD), we asked why the *N*-glycosylation-deficient 4-1BB was neither eradicated by ERAD nor became less stable. Since ERAD is initiated by recognizing abnormally glycosylated protein [23], we reasoned that the *N*-glycosylation-deficient 4-1BB does not contain the structural signature that triggers ERAD. However, we cannot rule out the possibility that *N*-glycosylated 4-1BB undergoes abnormal glycosylation and is therefore eliminated through ERAD. In addition to prior publications, which only reported the dimerized form of 4-1BB, we observed the existence of DTT-sensitive trimers and oligomers as well (especially for 2NQ), suggesting that additional free cysteine residues appeared due to the structural rearrangement caused by the abnormality of 4-1BB *N*-glycosylation. Another intriguing finding, which we were not able to outline yet, is that there are two distinct bands of 4-1BB 2NQ exhibited on SDS-PAGE. The major band which migrated faster is likely to be the Flag-tagged 4-1BB 2NQ protein, therefore, we presumed that the upper band refers to another type of modification that creates a molecular weight increase. The intensity ratio between the upper and lower band was significantly increased when the C121A mutation was included, indicating this specific modification is directly influenced by the thiol group on C121. In closing, further investigations should be conducted to uncover the difference between these two species.

In sum, we described a novel mechanism by which the organization and localization of 4-1BB are directed by *N*-linked glycan. Our findings manifested the correlation between the *N*-glycosylation status and membrane expression of the promising immunotherapy target 4-1BB. The individual N- glycosylation landscape could be harnessed as an indicator to predict the clinical efficacy of 4-1BB activation therapy.

## Data Availability

Not applicable.

## Supplementary Materials

The following are available online at https://www.mdpi.com/article/10.3390/cells11010162/s1, Figure S1: Confirmation of the PNGase F-mediated deglycosylation of 4-1BB; Figure S2: The overview of lentivirus vector design; Figure S3: Comparison of antibody detection of glycosylated and deglycosylated 4-1BB; Table S1: The relative percentage of permethylated N-linked glycans released from human 4-1BB.
