# Supplementary material for: N-Glycosylation Facilitates 4-1BB Membrane Localization by Avoiding Its Multimerization
Source: Cells. 2022 Jan 4;11(1):162. doi: 10.3390/cells11010162 (PMC8750214; doi:10.3390/cells11010162)
Supplement: Supplementary file 1 [file cells-11-00162-s001.zip › cells-1522386-supplementary.pdf]

Supplementary materials:

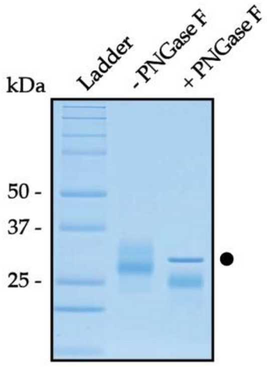

Figure S1. Confirmation of the PNGase F-mediated deglycosylation of 4-1BB. PNGase F-treated and untreated 4-1BB protein was separated on SDS-PAGE and visualized by coomassie blue staining. The band referring to PNGase F is indicated by the black circle.

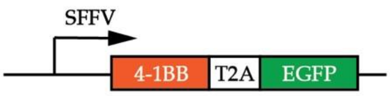

Figure S2. The overview of lentivirus vector design.

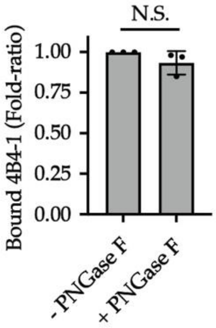

Figure S3. Comparison of antibody detection of glycosylated and deglycosylated 4-1BB. Same amount of plate-bound native and PNGase F-treated human 4-1BB protein was incubated with 4B4-1 at the concentration used for flow cytometry staining. HRP-conjugated anti-mouse secondary antibody was used for measuring bound 4B4-. N.S., not significant (two-tailed student's t-test).

Table S1. The relative percentage of permethylated N-linked glycans released from human 4-1BB. The masses of corresponding glycans are presented as [M+Na+] values. The structural determination of the N-glycans is based on the molecular weight, composition analysis via MALDI-MS derived data and follow the principles of the N-glycan biosynthesis pathway. Multiple possible structures might be assigned to one MALDI-MS peak.

No.	Mass (m/z) [M+Na+]	Percentage (%)	Possible structures	Glycan class
1	1416.6	1.22%	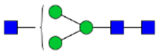	Hybrid
2	1579.7	4.91%	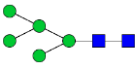	High mannose
3	1590.7	2.92%	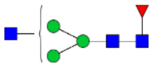	Hybrid [fucosylated]
4	1661.7	1.18%	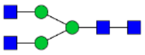	Complex
5	1783.8	0.21%	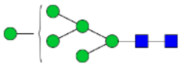	High mannose
6	1794.8	0.43%	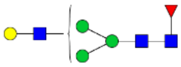	Complex [fucosylated]
7	1835.8	20.91%	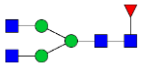	Complex [fucosylated]
8	1865.8	0.42%	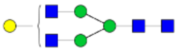	Complex
9	1987.9	0.23%	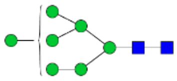	High mannose
10	2039.9	4.26%	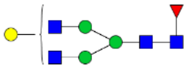	Complex [fucosylated]
11	2080.9	16.96%	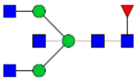 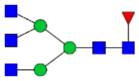	Complex [fucosylated]/ bisecting GlcNAc possible
12	2110.9	0.09%	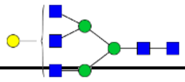 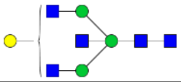	Complex/ bisecting GlcNAc possible
13	2214.0	1.30%	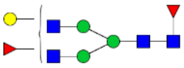	Complex [fucosylated]
14	2244.0	1.17%	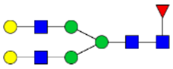	Complex [fucosylated]
15	2255.0	4.03%	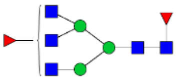 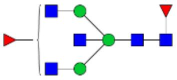	Complex [fucosylated]/ bisecting GlcNAc possible
16	2285.0	8.26%	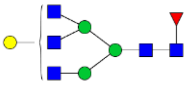 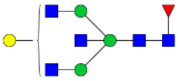	Complex [fucosylated]/ bisecting GlcNAc possible

17	2326.0	3.64%	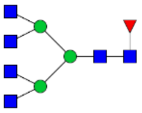 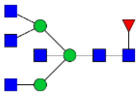	Complex [fucosylated]/ bisecting GlcNAc possible
18	2401.0	1.83%	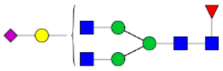	Complex [fucosylated, sialylated]
19	2418.1	0.43%	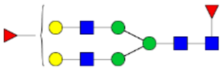	Complex [fucosylated]
20	2442.0	1.70%	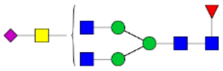	Complex [fucosylated, sialylated]
21	2459.0	6.04%	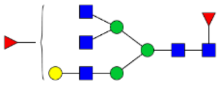 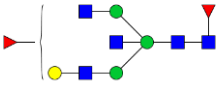	Complex [fucosylated]/ bisecting GlcNAc possible
22	2489.0	2.15%	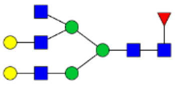 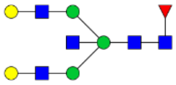	Complex [fucosylated]/ bisecting GlcNAc possible
23	2500.0	2.38%	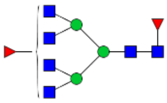 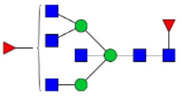 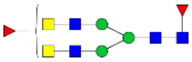	Complex [fucosylated]/ bisecting GlcNAc possible
24	2530.1	0.44%	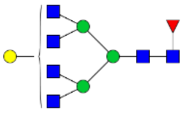 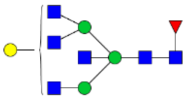	Complex [fucosylated]/ bisecting GlcNAc possible
25	2605.1	2.02%	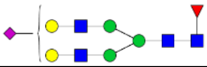	Complex [fucosylated, sialylated]
26	2633.1	1.36%	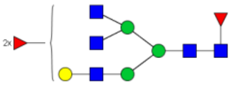 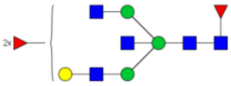	Complex [fucosylated]/ bisecting GlcNAc possible
27	2646.1	3.90%	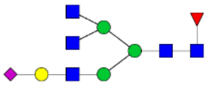 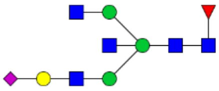	Complex [fucosylated, sialylated] / bisecting GlcNAc possible
28	2663.1	0.45%	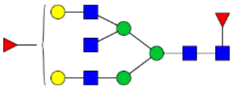	Complex [fucosylated]
29	2687.1	0.65%	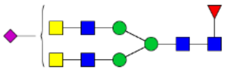	Complex [fucosylated, sialylated]
30	2779.2	0.18%	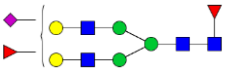	Complex [fucosylated, sialylated]
31	2820.2	2.71%	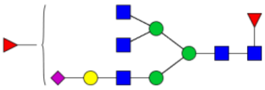 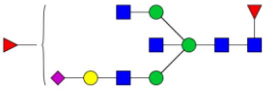	Complex [fucosylated, sialylated] / bisecting GlcNAc possible

32	2850.1	0.51%	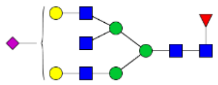 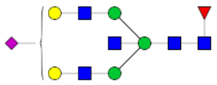	Complex [fucosylated, sialylated] / bisecting GlcNAc possible
33	2966.2	0.24%	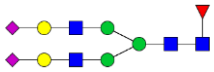	Complex [fucosylated, sialylated]
34	3007.2	0.88%	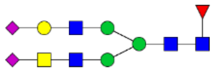	Complex [fucosylated, sialylated]
